# Mid-Infrared Generated Laser-Induced Grating Signals in Methane-Containing Gas Mixtures as Indicators of Composition, Pressure, and Temperature

**DOI:** 10.1177/00037028241230340

**Published:** 2024-02-19

**Authors:** Anna-Lena Sahlberg, Dina Hot, Zhongshan Li, Dimitrii Kozlov

**Affiliations:** 1 Combustion Physics, Department of Physics, 5193 Lund University, Lund, Sweden; 2 104659 Department of Optical Spectroscopy, Prokhorov General Physics Institute of the Russian Academy of Sciences, Moscow, Russia

**Keywords:** Nonlinear optical spectroscopy, laser-induced grating, LIG, gas sensing, methane detection, mid-infrared excitation

## Abstract

The present work is aimed at studying how spatially periodic modulations of the refractive index of the medium, i.e., laser-induced gratings (LIGs), generated in a gas mixture containing methane (CH_4_) by nanosecond pulses of resonant mid-infrared laser radiation, can be used to measure various gas parameters. It is investigated to what extent the temporal profiles of the LIG signals, recorded as the power of the diffracted by LIGs continuous wave probe radiation, are specific to the composition, pressure, and temperature of a selected buffer gas. This specificity is illustrated by the LIG signal profiles recorded in the experiments in different gas mixtures under various conditions. Experimental data show that large LIG signals can be obtained even in mixtures with CH_4_ concentrations as low as ∼100 parts per million due to the strong absorption of the excitation light and subsequent rapid, highly exothermic, and partner-dependent collisional energy exchange of the laser-excited molecules with the environment. These two factors ensure high LIG generation efficiency by a small number of CH_4_ molecules and high sensitivity of signal strength and profile to variations of gas parameters.

## Graphical abstract

**Graphical abstract:**
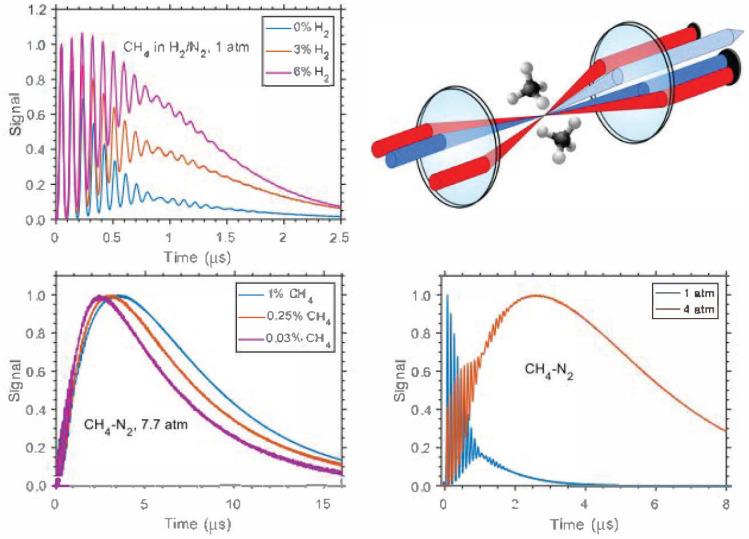
This is a visual representation of the abstract.

## Introduction

Advanced optical diagnostics using lasers provides possibilities for non-intrusive remote measurements of parameters of reactive gaseous media, in particular, during combustion.^1^ The nonlinear laser techniques of combustion diagnostics, based on four-wave mixing light–matter interactions,^2^ which employ, e.g., coherent anti-Stokes Raman scattering, degenerate four-wave mixing, and diffraction by refractive index gratings, enable accomplishing these measurements with a high-spatial resolution determined by small dimensions of the interaction volume of the laser beams as employed. In particular, the approach using spatially periodic modulations of the refractive index of the medium, or laser-induced gratings (LIGs),^3,4^ generated in a gas by two interfering beams from a nanosecond pulse laser, has demonstrated the potential to perform highly sensitive quantitative spatially and temporally resolved measurements. In this approach, the power of the continuous wave (CW) probe radiation diffracted by LIGs is detected as an informative signal. The signal strength, or the diffraction efficiency, at delay *t* after the pump pulse is determined by the square of the induced variation Δ*n*(*t*) of the refractive index *n* across the fringe of the grating.^3^ The parameters, such as gas temperatures^5–9^ and pressures,^10–13^ molecular number densities and species concentrations, fuel-to-air ratios,^14–18^ flow velocities,^19–23^ and collisional relaxation rates in electronic, as well as in rotational and vibrational energy transfer processes,^7,24–27^ have been determined from the LIG signal temporal profiles, *S*(*t*). The signals provided by the technique of LIGs and, especially, by that of laser-induced thermal gratings (LITGs), which is based on the deactivation and thermalization of resonantly excited probe molecules through collisions, allow a few parameters at a time to be derived and can be registered in a single laser shot.

To date, the physical background and the methodological aspects of LIG techniques employing nanosecond laser pulse pumping and CW laser probing in application to molecular gases are thoroughly developed and well understood in principle as a result of both theoretical and experimental investigations of numerous research groups worldwide during the last three decades (e.g., reviews by Kiefer and Ewart^2^ and Stampanoni-Panariello et al.^28^). At the same time, even simple polyatomic molecules have a multi-mode system of ro-vibrational levels and are characterized by various intramolecular ro-vibrational interactions and peculiarities of collisions between themselves and with the molecular environment. These interactions determine different pathways and rates of collisional internal energy transfer, and the amounts of the exchanged energy. Hence, the feasibilities of generation of thermal LIGs using these molecules in gas mixtures at elevated temperatures and pressures or in laboratory flames, of extraction of information about the medium from the LIG signal temporal profiles, as well as of the efficient employment of the technique in solving problems of practical importance, depend on a specific probe molecule and require separate study.

As far as the LIG technique for combustion diagnostics is concerned, the mid-infrared (mid-IR) spectral range of species excitation is of particular interest. This is because many combustion-relevant molecules, which play a key role in the reactions involved (e.g., hydrocarbons or water, H_2_O), lack easily accessible electronic transitions in the ultraviolet and visible (Vis) spectral ranges, but resonantly absorb radiation via strong and specific mid-IR ro-vibrational transitions in fundamental and combinational bands. Thus, these species can be selectively probed with a high sensitivity using narrowband frequency-tunable IR radiation. In addition, at resonant generation of LIGs using IR laser beams the signal temporal profiles can be efficiently recorded employing easily detectable visible read-out radiation.

Earlier, mid-IR generation of refractive index gratings in gas mixtures containing polyatomic molecules has been realized employing radiation of a pulsed fixed-wavelength CO_2_- laser. Small amounts of absorbing molecular species, such as ethylene (C_2_H_4_),^29–32^ sulfur hexafluoride (SF_6_),^29^ methanol (CH_3_OH),^30^ acetylene (C_2_H_2_), and ammonia (NH_3_),^32^ admixed to the buffer gases nitrogen (N_2_), argon (Ar), or helium (He), have been used to derive LIG signal temporal profile parameters and gas mixture properties, e.g., sound velocity and thermal conductivity, as a function of gas pressure, temperature, and composition, as well as to measure methane/air flame temperature. As a result, the diagnostic capabilities of the LITG technique with species excitation in the mid-IR range have been assessed.

Recently, LITGs generated by the mid-IR range 3.1 μm radiation were applied to the detection of C_2_H_2_, admixed in small portions to N_2_, air, carbon dioxide (CO_2_), and Ar,^33^ and to spectroscopy and detection of trace amounts of C_2_H_4_ in a gas cell, or of H_2_O, either in ambient air or in atmospheric C_2_H_4_/air flat flame.^34^ In this flame, resonant excitation of H_2_O molecules has been employed for local flame thermometry. Furthermore, in atmospheric methane/hydrogen/air flat flame the recorded mid-IR spectra of LITG generation efficiency allowed to perform flame temperature measurements employing strong transitions of hot H_2_O molecules.^35^

Methane (CH_4_) is the most thermally stable and the smallest saturated hydrocarbon. CH_4_ is involved in numerous gas reactions and is used for various technological and chemical processes as raw material and as common fuel. CH_4_ is one of the most important greenhouse gases and is also abundant in planetary objects. With growing interest in CH_4_, spatially and time-resolved quantitative diagnostics of CH_4_ is increasingly needed to advance the technologies and enlarge the efficiency of production and deep processing of hydrocarbon raw materials.

The highly symmetric tetrahedral CH_4_ molecule of the *T*_d_ point group with a specific tetrahedral structure of ro-vibrational levels has been thoroughly studied spectroscopically, as a model molecule with various intramolecular interactions within the so-called “polyads” of vibrational states,^36^ and in the processes of collisional *V*–*V*, *V*–*V*ʹ, and *V*–*T* relaxation,^37–43^ both in neat gas and in gas mixtures. The information obtained makes it possible to employ CH_4_ molecules for diagnostic purposes.

Spatially and temporally resolved highly sensitive optical diagnostics of either CH_4_ concentrations or gas parameters (by using CH_4_ as probe molecules present in small amounts) in reactive gas media in a broad range of temperatures and pressures is of great practical interest. It is these opportunities that the mid-IR generated LITG technique can provide.

Previous studies of CH_4_ using LITGs employed the excitation of the molecules by near-IR or visible laser radiation to high-lying ro-vibrational states via extremely weak overtone and combination band transitions. The dependence of optical pumping and of the collisional deactivation of these CH_4_ states on gas pressure has been investigated,^44^ the spectra of LITG generation efficiency have been recorded and analyzed,^45^ and ro-vibrational transition linewidths in these spectra have been measured.^46^ Generation of LITGs in CH_4_-containing gases employing resonant nanosecond pulse excitation of selected ro-vibrational levels of the ν_3_ vibrational state of CH_4_ molecules by mid-IR radiation at ≈3.2–3.4 μm enables realization of high sensitivity of the technique at diagnostics of CH_4_-containing mixtures, since in this case the strong dipole-allowed absorption transitions of the fundamental band, with large cross-sections, are involved.

In this regard, the goal of the present study is to further develop the technique of LITGs by using mid-IR pumping of different selected ro-vibrational transitions of the ν_3_ fundamental band of CH_4_ molecules, present as a small additive in various buffer gases, for diagnostics of gas mixtures relevant for combustion processes. In particular, this implies an investigation of the feasibility of finding relationships between the characteristics of the signal temporal profiles and such gas parameters as composition, pressure, and temperature, as well as the peculiarities of laser-excited CH_4_ collisional deactivation processes involved.

Hence, the study includes: (i) recording of temporal profiles of the LITG signals at mid-IR excitation of selected ro-vibrational transitions of the *P*-, *Q*-, or *R*-branches of the ν_3_ fundamental band of CH_4_ molecules in different buffer gases; (ii) comparison of the changes in specific profile characteristics at various conditions (CH_4_ concentrations, gas pressures, and temperatures) and in different buffer gases; and (iii) testing the feasibility to apply mid-IR generation of LITG signals for gas composition, pressure, and temperature diagnostics, as well as for deriving such parameters of these gases as thermal diffusivity and sound velocity.

## Experimental

### Materials and Methods

#### Laser Setup

The experimental setup employed in the experiments was described in detail earlier.^34^ Briefly, coherent pulsed narrowband tunable mid-IR pump radiation at around 3.3 μm (≈2 mJ, 3–4 ns, 10 Hz) was generated in a LiNbO_3_ crystal by difference frequency mixing of a Q-switched neodymium-doped yttrium aluminum garnet (Nd:YAG) laser radiation at 1064 nm with radiation at around 800 nm from a tunable dye laser, pumped by the frequency-doubled output of the Nd:YAG laser. The mid-IR radiation, at the frequency around 3000 cm^–1^, had a linewidth of ∼0.025 cm^–1^.^47^ The amplified IR beam, with a pulse energy of ≈1–3 mJ, was split by a 50/50 beamsplitter into two parallel pump beams, which were focused by a 300 mm focal length CaF_2_ lens and were aligned to cross in an interaction (probe) volume at an angle of ≈5.7°. The dimensions of the probe volume, close in shape to a prolate spheroid, were estimated to be ≈0.4 × 0.4 × 6 mm^3^, as calculated from the pump beam waist diameter in the crossing region and the crossing angle. The crossing parallel polarized pump beams were interfering with the probe volume with a fringe spacing of Λ ≈ 33 μm. Thermalization of the light energy absorbed by CH_4_ molecules in the probe volume resulted in a transient spatially periodic density pattern, which gave rise to a corresponding refractive index modulation known as a LITG.

Focused CW probe radiation of a frequency-doubled diode-pumped solid-state neodymium-doped yttrium orthovanadate (Nd:YVO_4_) laser (457 nm, 200 mW), with the beam waist in the focus adjusted to be equal to the transversal dimension of the probe volume, was directed to cross the probe volume at the Bragg angle, 0.38° to the optical axis, and to be diffracted by the LITG. The diffracted radiation was collimated by a 300 mm CaF_2_ lens and spatially and spectrally filtered employing an adjustable aperture and an interference filter (458 ± 10 nm). The temporal evolution of the power of this radiation, which shows the formation and decay of the grating, was recorded as a LITG signal by using a fast photomultiplier-based photosensor module (Hamamatsu, H6780-04) and a digital oscilloscope (LeCroy, WaveRunner 6100, 1 GHz). A He–Ne laser beam, spatially overlapped with the mid-IR beam, was used to facilitate the alignment of the invisible pump laser beams.

The measurements in mixtures of CH_4_ with different buffer gases at ambient pressure were performed in an open laminar gas flow, while the measurements in a wide range of pressures, from 0.2 atm up to 8 atm, were performed in a high-pressure gas cell equipped with sapphire windows. The cell could be filled with a desired amount of CH_4_ and of the selected buffer gas. The pressure in the cell was monitored by a pressure gauge (Special Instruments, Digima HP). Typical CH_4_ concentrations were in the range of 400–8000 parts per million (ppm), corresponding to CH_4_ partial pressures of ≈0.3–6 Torr under ambient conditions.

The measurements at different temperatures were carried out at ambient pressure in a slow laminar flow of N_2_ (7.5 L min^−1^) admixed with 1% of CH_4_. The flow was directed through a heating tube, which was an open T-shaped fused-silica gas tube, 15 mm in inner diameter, with 460 mm long gas inlet piece, and 160 mm long crosspiece. The tube body, wrapped by an electric heating wire and covered by thermal insulation, could be heated up to ≈800 K. The laser beams were aligned through the open-ended crosspiece. A thermocouple was inserted through the top of the heating tube to measure the temperature ∼2 mm above the probe volume, located in the middle of the crosspiece. Recently, the gas tube was used in the studies of LIGs in heated O_2_.^48^

### Ro-Vibrational Structure of the CH_4_ Molecule

The vibrational levels in CH_4_ molecules are characterized by four vibrational quantum numbers (υ_1_, υ_2_, υ_3_, and υ_4_), which correspond to the normal vibrational modes with the frequencies ν_1_ ≈ 2917 cm^–1^, ν_2_ ≈ 1533 cm^–1^, ν_3_ ≈ 3020 cm^–1^, and ν_4_ ≈ 1306 cm^–1^ (e.g., Yardley and Moore^39^). The vibrational states of CH_4_ molecules with accidental resonances are bound by strong intramolecular ro-vibrational interactions. The lower vibrational energy levels represent two groups of quasi-resonant states: the dyad, with ν_2_ ≈ ν_4_, and the pentad, where ν_1_ ≈ ν_3_ ≈ 2ν_2_ ≈ ν_2_ + ν_4_ ≈ 2ν_4_.^36^ Strong dipole-active ro-vibrational IR transitions of the fundamental asymmetric stretching vibration absorption band ν_3_ allow efficient resonant excitation of CH_4_ molecules into this vibrational state to be performed using mid-IR laser radiation. Selective generation of LITGs in CH_4_-containing gas mixtures by narrowband radiation at ≈3.3 μm is facilitated by a large number of ro-vibrational absorption lines of *P*-, *Q*-, or *R*- branches of the ν_3_ fundamental band of CH_4_ molecules.^49^ Each of these lines is split into tetrahedral (*T*_d_) *A*-, *E*-, or *F*-components corresponding to the molecules with different nuclear spins. In addition, each rotational level of the upper vibrational state, ν_3_, with the rotational quantum number *J*, is split by the Coriolis interaction into three sublevels with the pseudo-rotational quantum number *K* = *J*, *J* ± 1. The allowed optical transitions of the *P*-, *Q*-, and *R*-branches of the fundamental ν_3_ band correspond to the selection rules Δ*J* = *J* – *J″* = –1, 0, +1 (*J″* denotes the rotational quantum number in the ground vibrational state), and Δ*K* = 0. Note that the ro-vibrational transitions result in the excitation of the sublevels with the opposite parity: *A*_1_↔*A*_2_, *F*_1_↔*F*_2_, and *E*↔*E*.

The characteristic values of the absorption line strengths in the ν_3_ band at 295  K are ∼7 × 10^–20^ cm mol^–1^ (*P*(5) line component at ≈2968.7 cm^–1^). Hence, the absorption coefficient in the line center at characteristic 1 Torr pressure of CH_4_ in 1 atm CH_4_/N_2_ mixture (or at concentration 0.13%) is ≈0.016 cm^–1^. Therefore, only a negligible part of the pump radiation is absorbed under these conditions at the probe volume length of ≈6 mm.

The excited CH_4_ molecules participate in the collisional *R,V*–*R*′*,V*′ and *R,V*–*T* energy exchange with the medium. In N_2_ at 1 atm gas pressure and ambient temperature binary gas kinetic collisions occur on average in τ_c_ ≈ 0.09 ns at a collision-free path of only ≈0.054 μm. The energy exchange results in the modulation of the gas density due to small spatially periodic local temperature variations and, hence, in a periodic variation of the refractive index, i.e., in the formation of a LITG. The rates of relaxation and energy exchange for various CH_4_ vibrational states were being extensively studied both in neat gas and in mixtures with rare and molecular gases.^37–43,50,51^

## Results and Discussion

To study the manifestation of gas composition, pressure, temperature, and collisional deactivation of the laser-excited CH_4_ molecules in the LITG signal temporal profiles specific to the gas mixtures employed, the gratings were generated using one or a group of close *T*_d_ components of a selected *P*-, *Q*-, or *R*- branch ro-vibrational transition of the ν_3_ fundamental band of CH_4_. The molecules were admixed, at low concentrations, to different gases (e.g., Ar, N_2_, hydrogen (H_2_), and CO_2_), and the temporal profiles of the LITG signals were recorded at different mixture compositions, pressures, and temperatures.

### Signal Temporal Profiles and Their Features

**Figure 1.**
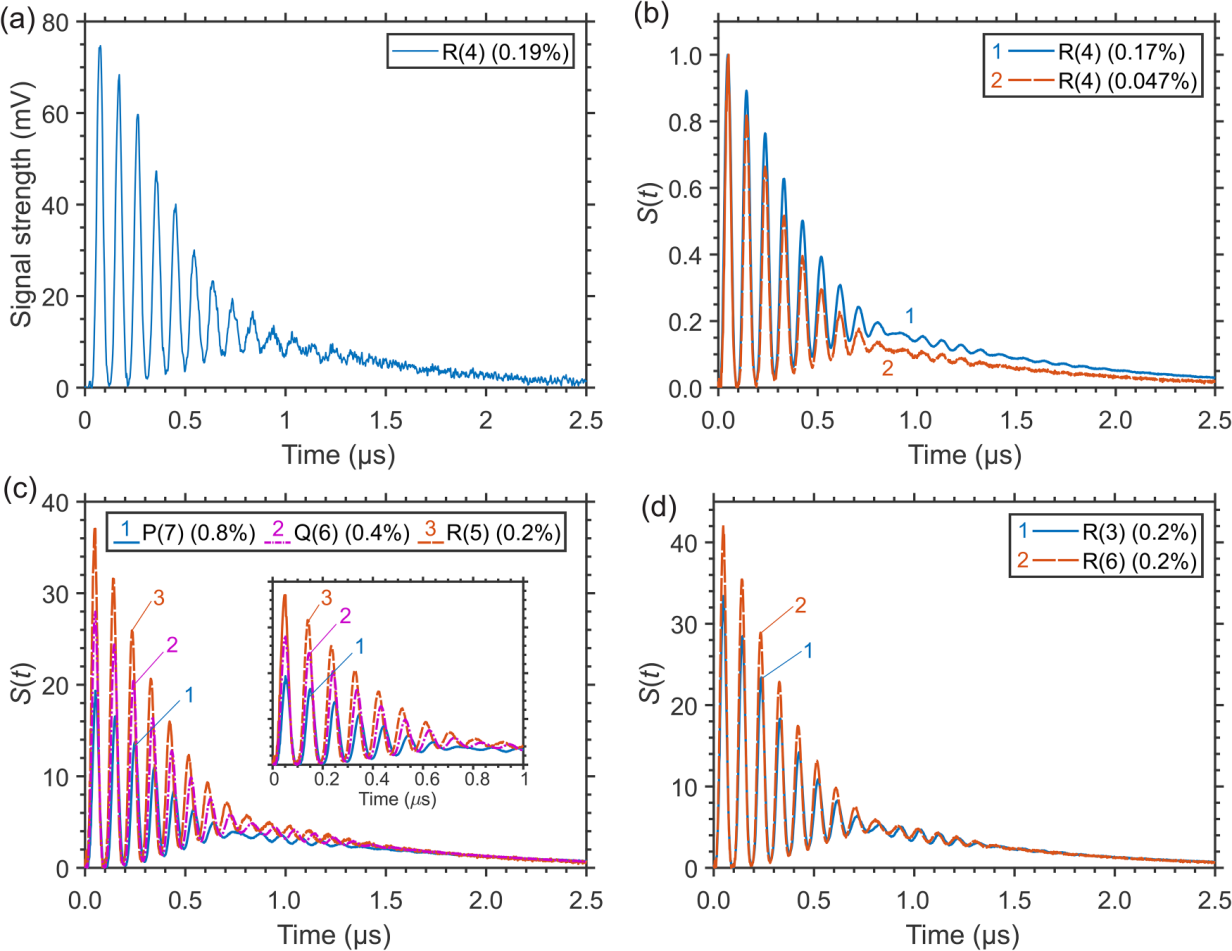
Laser-induced thermal grating (LITG) signals in CH_4_/N_2_ mixtures at 1 atm and *T* = 299 K: (a) a single-shot signal and (b) accumulated signals at CH_4_ excitation through the *R*(4) transition; the profiles in (b) are normalized by the amplitude of the first oscillation peak; (c) accumulated signals at CH_4_ excitation to the *J* = 6 upper level via *P*(7), *Q*(6), and *R*(5) transitions; the inset shows the oscillation peaks more clearly; (d) accumulated signals at CH_4_ excitation to different upper rotational *J* = 4 and *J* = 7 levels through *R*(3) and *R*(6) transitions; the profiles in (c) and (d) are normalized by their strengths at 2.2 μs.

As an example, typical LITG signals recorded at ambient temperature *T* = 299 K in an open-air flow of 1 atm CH_4_/N_2_ mixtures with different small CH_4_ concentrations are presented in Figures 1a and 1b. Figure 1a shows a single-shot signal detected at 0.19 vol% of CH_4_ (partial pressure 1.40 Torr), which shows a reasonably high signal-to-noise ratio. Figure 1b presents similar signals averaged over 500 single laser shots and obtained at maximal 0.17 and minimal 0.047 vol% of CH_4_ (i.e., partial pressures 1.30 and 0.35 Torr, respectively). The corresponding gratings were generated at resonant excitation of CH_4_ molecules via the multiplet of closely spaced four *T*_d_ components of the *R*(4) transition at 3067.3 cm^–1^.^49^ The initial part of the LITG signal profiles is represented by a non-stationary contribution with characteristic oscillations. Since the signal strength drops sharply with the reduction of CH_4_ concentration, for comparison, the profiles in Figure 1b are normalized by the amplitude of the first peak of the oscillations. The oscillations, with the period *T*_a_, result from the buffer gas density modulation in the form of a standing acoustic wave with the frequency Ω_a_ = 2π/*T*_a_. The value of Ω_a_ is defined by the fringe spacing Λ and the adiabatic sound velocity υ_s_ in the gas: Ω_a_ = 2πυ_s_/Λ.^3,4,25^ The standing acoustic wave is generated if the ro-vibrational energy exchange of the laser-excited CH_4_ molecules with the environment due to collisions is rapid (“instantaneous”), i.e., if the relation 1/τ_c_ >> Ω_a_ for the inverse average time between the respective collisions and the acoustic frequency is valid.^4,25^ The amplitude of the first signal oscillation peak is determined by the squared product of the absorption coefficient and the amount Δε_i_ of the instantaneously exchanged energy per molecule. While the signals in Figure 1b are normalized by the amplitude of this peak, the actual signal strengths are proportional to the square of the absorption coefficients and therefore the corresponding CH_4_ concentrations. This means that the recorded signal at 0.047% is actually ≈16 times weaker than that at 0.17% at the same pump laser energy. At the same time, the signal profiles do not contain a non-resonant acoustic contribution caused by adiabatic electrostrictive gas compression and are characterized by a doubled oscillation frequency of 2Ω_a_.^4^

In LITG excitation volumes with large transverse dimensions, the decay of the acoustic wave amplitude in the gas is due to viscosity and heat conduction and is characterized by the acoustic time τ_a_:^3,4,25^

(1) $$\tau_{a}=2\cdot {\left(\frac{\Lambda }{2\pi }\right)}^{2}\cdot {\left\{\frac{1}{\rho }\left[\frac{4}{3}\mu +(\gamma -1)\cdot \frac{\kappa }{c_{p}}\right]\right\}}^{-1}$$

where Λ is the fringe spacing of the LIG, ρ is the spatially homogeneous mass density, μ is the dynamic viscosity, γ = *c*_p_*/c*_v_ is the ratio of specific heats at constant pressure and volume, and κ is the thermal conductivity of the gas. However, the generation of LITGs by focused pump beams with small transverse dimensions causes the faster decay of the standing acoustic wave amplitude since the two counter-propagating acoustic wave packets leave the excitation volume. For the Gaussian beam profiles the corresponding acoustic transit time, τ_tr_, can be characterized by the value $w_{0}/\sqrt{2}\upsilon_{s}$ , where *w*_0_ is the 1/e*^2^* level waist radius of the pump beams in the crossing area.

The small difference in the peak-normalized profiles in Figure 1b is observable at delays *t* = 0.7–2.5 μs after the pump pulse, when the stationary contribution to the LITG signal caused by relatively slow vibrational relaxation^4,25^ already begins to appear. This difference is due to almost a fourfold excess of CH_4_ molecules at 0.17% concentration compared to 0.047%. However, the dependence of the LITG signal profile on CH_4_ partial pressure in this case is weak. One can notice that at lower CH_4_ concentrations the stationary contribution at 0.7–2.5 μs decreases relative to the contribution from the rapid energy exchange (amplitudes of the peaks in the initial part of the signal). This might be due to a lower *V*–*V*′ exchange rate and a smaller “fast” energy transfer of the excited CH_4_ molecules because of the reduction of the number of their collisions with other CH_4_ molecules in the ground state. In other words, it can be assumed that this is a manifestation of an increase, with increasing CH_4_ concentration, in the contributions of the finite-rate (“fast” and “slow”) collisional relaxation to the LITG signal profile due to both a few-stage energy transfer within the manifold (ν_3_, ν_1_, ν_2_ + ν_4_, 2ν_4_, and ν_4_) and the deactivation of the ν_4_ state. These processes determine the stationary gas density modulation responsible for the contribution to the signals observed at *t* > 0.7 μs. This stationary modulation decays due to heat conduction with time τ_th_:^4,25^

(2) $$\tau_{\mathrm{th}}={\left(\frac{\Lambda }{2\pi }\right)}^{2}\cdot {\left(\frac{\kappa }{\rho c_{p}}\right)}^{-1}$$

defined by the grating spacing Λ, mass density ρ, specific heat at constant pressure *c*_p_, and thermal conductivity of the gas κ.

Along with the processes of collisional deactivation and energy exchange, the induced additional (non-equilibrium) spatially periodic population of the vibrationally excited CH_4_ molecules is destroyed by their mass diffusion across the fringes with the time τ*_D_*:^4,25^

(3) $$\tau_{D}={\left(\frac{\Lambda }{2\pi }\right)}^{2}\cdot \frac{1}{D}$$

where *D* is the state-specific mass diffusion coefficient at a given gas density, *D* ∼ 1/ρ.

The values of the characteristic times τ_a_, τ_th_, and τ*_D_* are of the same order in a gas. They all scale as Λ^2^ and are roughly proportional to mass density (molecular number density) of the gas, or gas pressure at a given temperature. Adding buffer gas to a small amount of resonantly absorbing species, and hence increasing gas density, slows down mass diffusion and stimulates collisional deactivation of the excited molecules. As a result, more and more of them exchange their energy spatially in phase making the thermal contribution to the LIG signal stronger. At low concentrations of CH_4_, used in the experiments, the values of the adiabatic sound velocity υ*_i_*, and the times τ_a_, τ_th_, τ*_D_*, and τ_tr_ are determined by the parameters of the buffer gas, that is, N_2_ in the case of the signals in Figure 1. Here, at Λ ≈ 33.2 μm, the respective values at ambient temperature and pressure are estimated to be: υ_s_ = 353 m s^–1^, *T*_a_ = 0.094 μs (Ω_a_ = 67 μs^–1^), τ_a_ = 1.35 μs, τ_th_ = 1.27 μs, and τ*_D_* ≈ 1.18 μs. Hence, the generated acoustic waves are developing under conditions of weak damping, which means *T*_a_/2π << τ_a_, τ_th_.^4,25^ In addition, strong oscillations caused by the energy release indicate that its characteristic time τ_c_ is small and satisfies the above-mentioned condition: Ω_a_·τ_c_ << 1, i.e., τ_c_ << 0.015 μs in the case of the signals in Figure 1. Note that with focusing providing pump beam waist diameter 2*w*_0_ ≈ 400 μm, the transit time τ_tr_ is estimated to be about 0.54 μs. Thus, in this case τ_tr_ is noticeably smaller than τ_a_ and has a stronger effect on the evolution of the LITG signals already at pressures above 0.5 atm.

The normalized signal profiles in CH_4_/N_2_ mixtures in the cell at 1 atm and temperature of 299 K are noticeably (but insignificantly) dependent on either the branch of the resonant transition employed or the value of *J* itself within the same branch, as demonstrated by the accumulated signals in Figures 1c and 1d. Figure 1c presents the signals at CH_4_ excitation to the same upper rotational level *J* = 6 through the *P*(7) (*F*_1_–*F*_2_), *Q*(6) (*E–E, F*_2_–*F*_1_), and *R*(5) (*F*_2_–*F*_1_) transitions characterized by different sign and amount of the rotational energy exchange (at 5.7, 2.9, and 1.4 Torr CH_4_, respectively). Figure 1d shows the signals when CH_4_ is excited to different upper rotational levels *J* = 4 and *J* = 7 via the *R*(3) (*A*_2_–*A*_1_, *F*_2_–*F*_1_*, F*_1_–*F*_2_) and the *R*(6) (*A*_1_–*A*_2_, *F*_2_–*F*_1_, *A*_2_–*A*_1_) transitions characterized by the same sign, but different amount of the exchanged rotational energy (at 1.4 Torr CH_4_). The profiles in Figures 1c and 1d are normalized by their strengths in the signal tail (at delay *t* = 2.2 μs), which in this case can be regarded as a normalization by the number of excited molecules. The noticeable very small difference in the oscillation periods is due to the difference in the excitation wavelengths. Note that the observed similarity of the LITG signal profiles contrasts with the distinct difference of those observed at laser excitation of ro-vibronic transitions in *P*- or *R*-branches of the O_2_ A-band at near ambient pressure (see the analysis by Hubschmid and Hemmerling^24^). In O_2_, the signal profile difference is due to, respectively, negative (Δε_R_^P^ ≈ –2*B*·(*J* + 1)) or positive (Δε_R_^R^ ≈ 2*B*·*J*) amount of the rotational energy Δε_R_, which is rapidly, in a few collisions, exchanged with the medium. At excitation of CH_4_, the profile resemblance in the CH_4_/N_2_ mixture is apparently the consequence of the rapid release of a larger amount of vibrational energy due to collisional vibrational energy transfer (VET) in inter-mode *V*–*V′* transitions that dominates the LITG signal formation. This is confirmed by the signal profile at CH_4_ excitation within the *Q*-branch (see Figure 1c), for which Δε_R_^Q^ ≈ 0.

**Figure 2.**
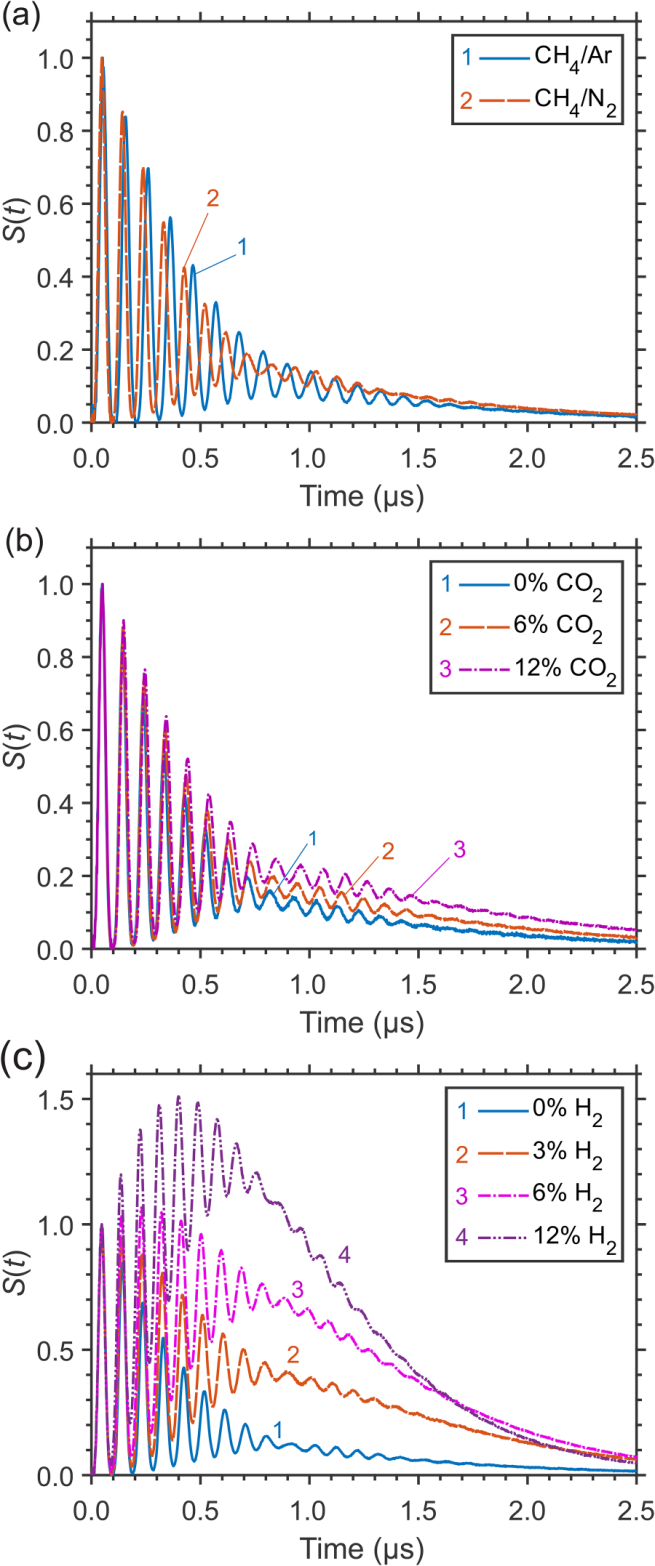
Normalized LITG signals at 1 atm and ambient temperature in the cell: (a) in CH_4_/Ar and CH_4_/N_2_ mixtures with 0.2% CH_4_; (b) in CO_2_/N_2_ mixtures with 0.3% CH_4_, at increasing CO_2_ concentration; (c) in H_2_/N_2_ mixtures with 0.2% CH_4_, at increasing H_2_ concentration.

The LITG signal profiles are strongly dependent on the environment of the absorbing molecules. Due to the specific molecular and transport properties of various buffer gases, as well as changes in collisional VET mechanisms and rates of excited CH_4_ in these gases, the signals at a given pressure have different profiles, which indicates different sensitivity of these profiles to gas composition. The examples of LITG signals obtained in the cell containing a small amount of CH_4_ in several buffer gases at 1 atm and ambient temperature are presented in Figure 2. All the profiles are normalized by the amplitude of the first oscillation peak.

In Figure 2a, the signals generated using the *R*(4) transition of 0.2% CH_4_ molecules (at partial pressure 1.4 Torr) either in an inert gas Ar or in N_2_ are compared. Monatomic buffer gas provides the simplest collisional relaxation mechanism of laser-excited CH_4_ molecules. Clearly visible *T*_a_ difference in the signal profiles at CH_4_ excitation via the same transition corresponds to the 9% difference of the sound velocities in Ar and N_2_. Note that the profiles in Figure 2a practically do not differ in the strength of the stationary contribution at delays of more than 0.7 μs. This indicates that the presence of vibrational structure in N_2_ molecules as quenchers has little effect on collisional relaxation in CH_4_. At the same time, in CH_4_/Ar mixtures, there is practically no dependence of signal profiles on the CH_4_ partial pressure, in contrast to the case of CH_4_ in N_2_ (shown in Figure 1b). A comparison of the signals generated in the CH_4_/Ar mixture via different branches and *J*-transitions of the ν_3_ band at the same CH_4_ concentrations, normalized by their strengths in the tail at 2.2 μs, shows an even weaker profile dependence on the excitation branch and *J* value compared to that in N_2_ (as shown in Figures 1c and 1d). This may indicate that rapid rotational energy transfer (RET) and VET of CH_4_ molecules due to collisions (predominantly) with Ar atoms proceed more slowly compared to the case with N_2_ molecules. These facts indicate a more complex collisional relaxation mechanism of CH_4_ in a gas of even simple homonuclear molecules, compared to the mechanism of relaxation occurring in an inert monatomic gas.

The examples of LITG signal profiles related to even more efficient “fast” and “slow” collisional relaxation of CH_4_ molecules at 1 atm within the manifold of the vibrational states (ν_3_, ν_1_, ν_2_ + ν_4_, 2ν_4_, and ν_4_) and from the ν_4_ state, and/or to more complicated relaxation schemes in gas mixtures are shown in Figures 2b and 2c. There, the normalized signals in CO_2_/N_2_ and H_2_/N_2_ gas mixtures of different compositions, with a small percentage of CH_4_ (at partial pressures 2.0 and 1.4 Torr, respectively), are presented. The mixtures were continuously and slowly flowing through the gas cell. The transitions used for CH_4_ excitation were *R*(2) (*F*_2_–*F*_1_, *E*–*E*) in CO_2_/N_2_ and *R*(4) in H_2_/N_2_. The addition of such quenchers as CO_2_ or H_2_ to N_2_ significantly changes the signal profiles, increasing the contribution to the part that is determined by the “slower” relaxation processes. In particular, adding H_2_ causes the “slower” relaxation to occur at even earlier times than in neat N_2_, i.e., at *t* ≥ 0.3–0.5 μs (compare Figures 2a and 2c). Note that in H_2_ *V–T* relaxation and energy release appear to be more efficient than in CO_2_ and N_2_.

The noticeable monotonic variation of the signal oscillation periods *T*_a_ with the concentration of CO_2_ in Figure 2b (*T*_a_ increases) or with that of H_2_ in Figure 2c (*T*_a_ decreases) is due to the dependence of the sound velocity on gas composition. The LITG signal strength also varies with the quencher concentration.

**Figure 3.**
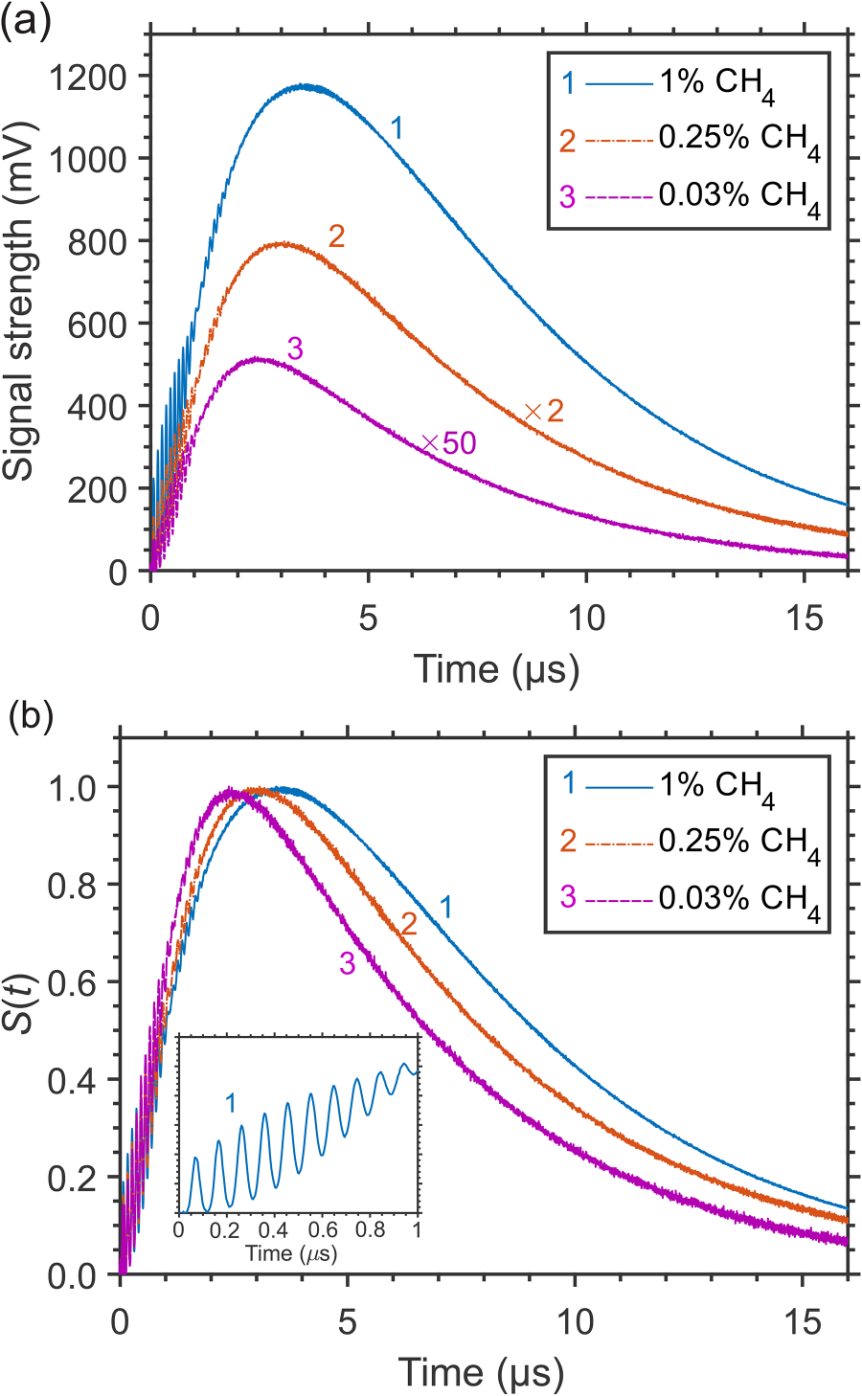
LITG signal profiles at CH_4_ excitation through the *R*(4) transition in N_2_ at 7.7 atm and *T* = 299 K at different CH_4_ concentrations in the cell: (a) normalized by the squared pump laser energy (the signals at lower concentrations are multiplied by factors of 2 and 50, respectively, to be visible at the same scale); (b) normalized by the amplitude of the hump. The inset more clearly shows the characteristic oscillation peaks at the steep front slope of the signal profiles.

In CH_4_/N_2_ mixtures at elevated pressure, the release of large quantum ν_4_ of vibrational energy at the stage of “slow” relaxation becomes more efficient and more pronounced, due to faster *V–T* relaxation and slower mass diffusion with increasing pressure, and results in a “hump” on the signal tail, as shown in Figure 3a. There, the signals at decreasing CH_4_ concentrations (partial pressures) in 7.7 atm N_2_ gas, with a hump at *t* ≈ 3–4 μs, are presented. The signal profiles qualitatively differ from those at 1 atm in Figure 1, having a relatively smaller contribution of the “instantaneous” and “fast” release of the vibrational energy. However, the rapid decrease of the signal strength at lower CH_4_ concentrations is similar to that observed at 1 atm. Figure 3b presents the signals in Figure 3a normalized by the hump amplitude value, demonstrating still a relatively weak dependence of the LITG signal profile on the CH_4_ concentration due to self-collisions also at 7.7 atm, the shift of the hump maximum and the increase of its width with CH_4_ concentration being the main changes.

Modeling of the signal temporal profiles in CH_4_-containing gas mixtures aimed at understanding the mechanisms of their formation and specificity, as well as at deriving information on the parameters of the gas medium, can be carried out using the approach outlined in detail by Hemmerling and Kozlov^25^ and Hubschmid^27^ in application to LIG signals obtained in neat O_2_ and O_2_/CO_2_ mixtures. This approach is similar to that used later by Kozlov and Radi^44^ to model the signals resulting from high vibrational excitation of CH_4_ molecules. It is based on a simplified VET kinetic scheme employing the rates of non-radiative ro-vibrational transitions known from the literature and neglecting the deactivation due to the radiative transitions from the ν_3_, ν_2_ + ν_4_, 2ν_4_, and ν_4_ vibrational states with the rates being at least two orders of magnitude lower than those of the vibrational relaxation.^39^ However, it is worth noting that the information on the collisional relaxation pathways and molecular transition rates, necessary for modeling the evolution of LITGs in various molecular gases, is not always available.

### Signal Strengths and Profiles, and Gas Composition

To evaluate the sensitivity of various LITG signal parameters to CH_4_ concentration, *x*, at various pressures, and to assess the limits of its efficient detection in the experiment, the excitation of CH_4_ molecules in N_2_ via the strong *R*(4) transition of the ν_3_ band has been employed. The *R*(4) absorption line strength is the maximum at a gas temperature close to the ambient one, while, e.g., in the range of 1500–1800 K characteristic for CH_4_ pyrolysis, it is only ∼1.5 times smaller than that of the *R*(8) and *R*(9) lines, which are the strongest CH_4_ absorption lines at those temperatures.

At ambient pressure of a CH_4_/N_2_ mixture, with the typical LITG signal profiles as presented in Figure 1, it is straightforward to perform the concentration measurements using the strong change in the signal strength depending on CH_4_ concentration *x*. For this case, the sensitivity of the technique and the assessment of the detection limit of the experiment have been studied at various *x* decreasing in the range 0.17–0.047%.

As explained above, the amplitude of the largest, first peak of signal oscillations *I*_1_, normalized by the square of the excitation pulse energy, scales as the square of the number of the absorbing molecules, or their concentration, that is, *I*_1_ ∼ *x*^2^. Hence, CH_4_ concentrations can be derived employing the *I*_1_(*x*) dependency calibrated in one point. The detection limit of the setup when determining low concentrations using this approach was estimated, based on the values of *I*_1_ and signal-to-noise ratios ∼100 in the single-shot signal (Figure 1a) and ∼1600 in the accumulated signal (Figure 1b, 0.17% CH_4_), as ∼0.02% (200 ppm), and ∼0.005% (50 ppm), respectively, at ambient N_2_ pressure and temperature. The noise level was estimated as an average amplitude of random fluctuations of the signal at the tail of its profile. The detection limit can be further reduced if not the amplitude *I*_1_, but the integral of the LITG signal profile over a definite interval of delays *t* is employed. Note, for comparison, that the background level of CH_4_ in the atmosphere is 1859 ± 2 parts per billion (ppb)  ≈ 2 ppm.

**Figure 4.**
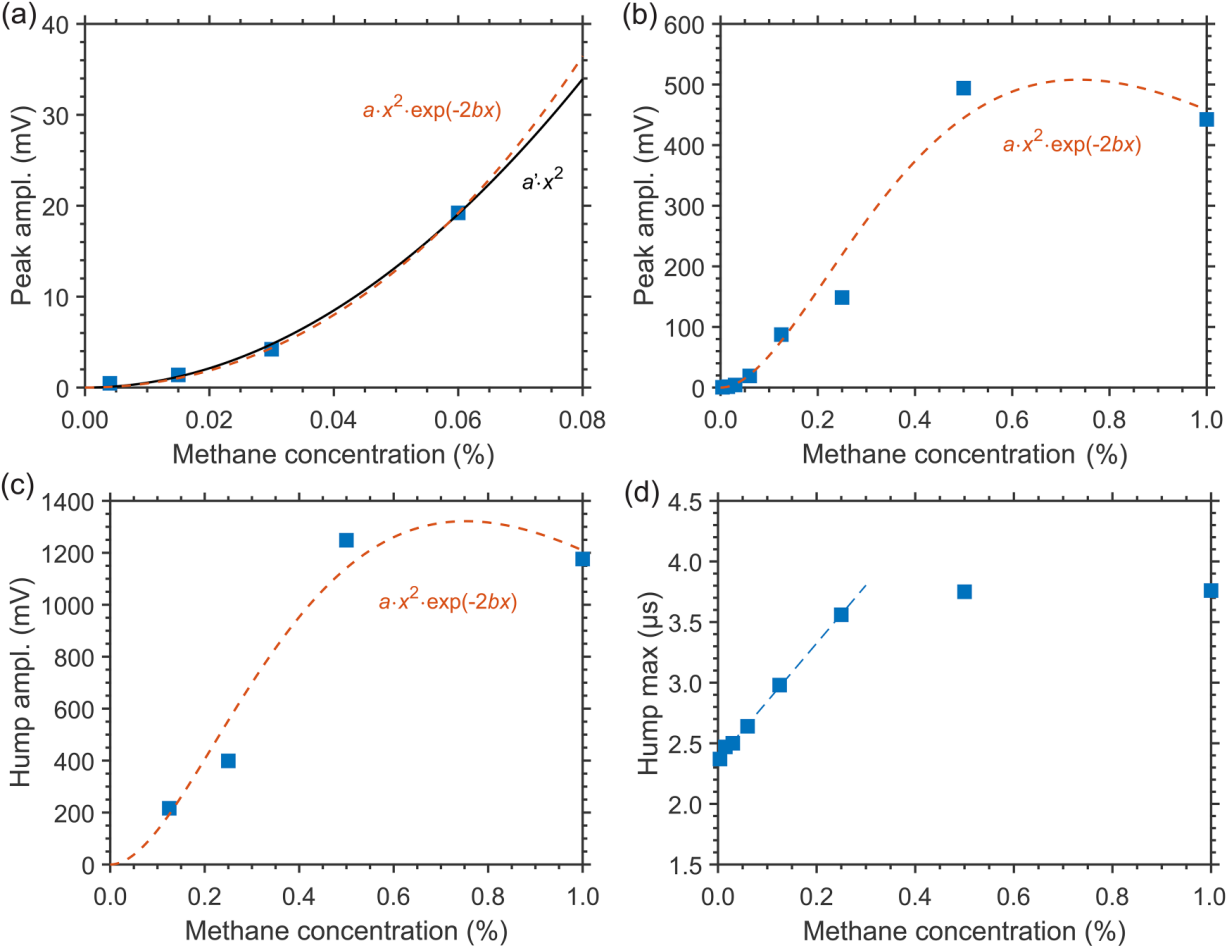
LITG signal profile characteristics at CH_4_ excitation through the *R*(4) transition in the cell with N_2_ at 7.7 atm and *T* = 299 K as a function of CH_4_ concentration: (a) the amplitude of the first oscillation peak at low concentrations (black solid line: fitted quadratic dependency; red dashed line: fitted dependency using the Beer–Lambert law); (b) the first oscillation peak and (c) the hump amplitude at larger concentrations (red dashed line: fitted dependency using Beer–Lambert law); (d) position (μs) of the signal hump maximum (blue dashed line: fitted linear dependency). The solid squares represent the experimental values.

For CH_4_/N_2_ mixtures at higher pressure, the CH_4_ concentration dependency of the LITG signals has been investigated in the wider range of *x* = 0.0058–1% at 7.7 atm of N_2_, with the signal profiles similar to those presented in Figure 3. For these measurements, both the first oscillation peak and hump amplitudes (*I*_1_ and *I*_h_) have been employed. The obtained dependencies *I*_1_(*x*) and *I*_h_(*x*) are shown in Figures 4a–4c.

At low CH_4_ concentrations (*x* ≤ 0.1%), when the absorption of the pump radiation in the gas is weak, the growth of the oscillation peak amplitude *I*_1_ is quadratic with *x*, as shown in Figure 4a by the curve *I*_1_ = *a'*·*x*^2^ fitted to the experimental data, with the scaling factor *a*′ as a parameter.

At larger CH_4_ concentrations (*x* > 0.1%) the absorption increases, so that the signal growth can become “saturated,” which leads to a decrease in measurement sensitivity, as shown in Figures 4b and 4c for the *I*_1_ and *I*_h_ amplitudes, respectively, due to the reduction of the pump beam intensities on the way to the probe volume inside the gas cell. This behavior can be modeled incorporating the Beer–Lambert law as *I*= *a*·exp(–2*bx*)·*x*^2^, where *b* is the pressure- and temperature-dependent attenuation coefficient multiplied by the optical path length. Fitting of the experimental data gives the values *b* = 1.25 ± 0.13 for *I*_1_ and *b* = 1.38 ± 0.20 for *I*_h_, similar within errors and in reasonable agreement with the estimate *b* ≈ 1.4 taking into account the absorption line strength, the buffer gas pressure, and the optical path length of the mid-IR laser beams through the gas cell (about 30 mm). Note that the curve *I*_1_ = *a*·exp(–2*bx*)·*x*^2^, fitted to the data in Figure 3a, with fixed *b* = 1.25, and plotted there, practically coincides with that of the derived quadratic dependency. This is also valid for the low-concentration data at ambient pressure represented by Figure 1a. Thus, during the measurements of low CH_4_ concentrations (*x* < 0.1–0.15%) the effects of pump radiation absorption can be neglected, while care should be taken at the higher values of *x*. To perform reliable measurements with high sensitivity in this case, one must tune to a weaker absorption line to reduce the attenuation coefficient and the effect of signal saturation.

The CH_4_ detection limit at 7.7 atm N_2_ pressure and ambient temperature was estimated, based on the values of *I*_1_ and *I*_h_ of single-shot and accumulated signals at 0.13% CH_4_ concentration and signal-to-noise ratios for these signals. The detection limits derived using the amplitudes of the oscillation peak were found to be comparable to those obtained for atmospheric pressure. At the same time, the use of the higher amplitudes of the hump (see Figure 3a) provided lower detection limits of ∼0.004% (40 ppm) and ∼0.0007% (7 ppm) for the single-shot and accumulated signals, respectively.

Though at ambient pressure the dependence of the LITG signal profile on CH_4_ partial pressure and concentration *x* is weak and hardly observable, as seen in Figure 1b, at higher N_2_ pressures it becomes more pronounced, as demonstrated by Figure 3b, and discussed above, due to faster CH_4_-defined *V*–*V*′,*T* collisional relaxation, with large ν_4_ quantum of the vibrational energy release, and slower CH_4_ mass diffusion. These factors both increase the stationary density modulation and the corresponding contribution to the LITG signal profile at *t* > 1 μs. In particular, the position of the signal hump maximum in Figure 3b shifts to larger delays linearly with *x* in the range 0% < *x* < 0.25%, as presented in Figure 4d. This shift can thus be additionally used for independent CH_4_ concentration measurements. However, as seen in Figure 4d, the shift tends to saturate at 0.25% < *x* < 1% for the *R*(4) absorption line. Therefore, under these conditions, the position of the hump maximum is a useful indicator of CH_4_ concentration only at levels below 0.25%.

**Figure 5.**
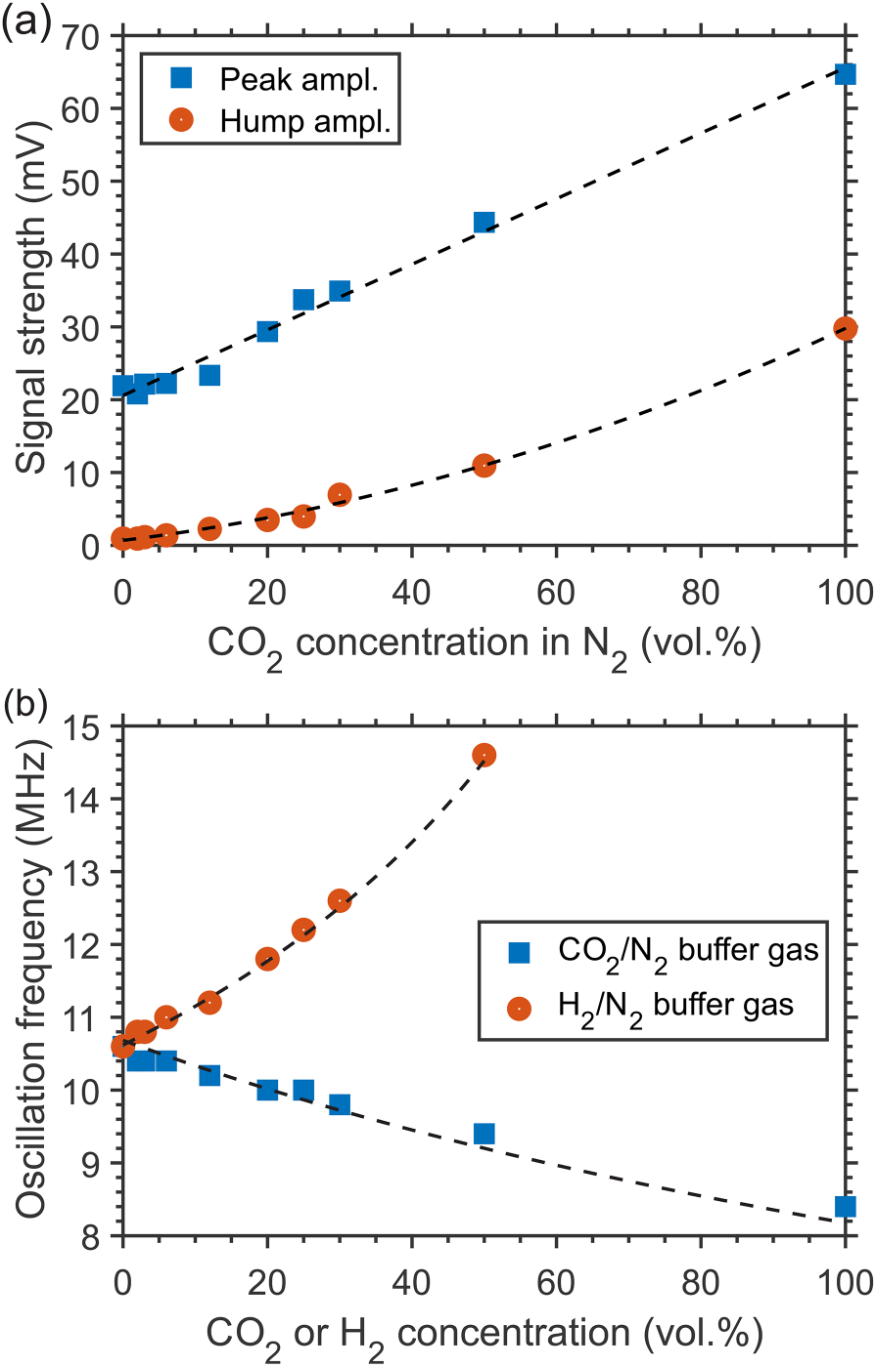
Variations of LITG signal profile characteristics with a binary mixture composition: (a) LITG signal strength in atmospheric pressure CO_2_/N_2_ (0.3% CH_4_) mixtures versus CO_2_ concentration (blue squares: the first oscillation peak amplitude; dashed line: linear fit; red circles: the strength of the stationary contribution at *t* = 2 μs; dashed line: quadratic polynomial fit); (b) the oscillation frequency versus component concentration for the signals in atmospheric pressure CO_2_/N_2_ (0.3% CH_4_) and H_2_/N_2_ (0.2% CH_4_) mixtures; the solid symbols represent the values derived from the signal profiles, the dashed lines show the results of calculations using Eq. 5.

As presented in Figures 2b and 2c for the mixtures CO_2_/N_2_ and H_2_/N_2_ at 1 atm and ambient temperature, the composition of a binary buffer gas mixture, with the addition of a negligible number of absorbing CH_4_ molecules, defines specific features of the generated LITG signal profiles, which demonstrate high sensitivity to the variation of the buffer gas composition. This allows the concentration *y* of one of the binary mixture components to be defined, at a given gas temperature and pressure. As has been shown above for CH_4_/N_2_ mixtures, the concentrations can be derived (after calibration) from the signal strength defined by mixture-specific relaxation processes of the excited CH_4_ molecules. This possibility is illustrated by Figure 5a. There, changes of the first oscillation peak amplitude or the stationary contribution strength at *t* = 2 μs of the LITG signal at CH_4_ excitation via the *R*(2) transition in atmospheric pressure CO_2_/N_2_ gas mixtures, with 0.3% CH_4_, are presented as a function of CO_2_ concentration. The fitted linear or quadratic polynomial dependencies, shown here as the dashed lines, can be used as the calibration curves.

Another possibility of measuring the concentration of components in a binary buffer gas mixture under isothermal conditions is to use the LITG signal temporal profile, instead of the signal strength, which depends on the shot-to-shot fluctuating pump laser intensity. This possibility is demonstrated in Figure 5b. Previously, a similar approach was implemented in real binary mixtures employing electrostrictive^28^ or thermal^16^ LIGs. Here, the oscillation frequency *f* = 1/*T*_a_ of LITG signals actually in ternary mixtures is employed. This frequency is determined by the temperature-dependent adiabatic sound velocity in the mixture and derived using the Fourier transform to the temporal profiles. The analysis is applied to the signals registered in CO_2_/N_2_ and H_2_/N_2_ mixtures with a small amount of CH_4_ admixed (see Figures 2b and 2c). For a real binary mixture of ideal gases, the dependency *f*(*y*) ~ υ_s_(*y*) at a given temperature can be described as:

(4) $$\begin{array}{rl} & f(y)=\\ & \;f_{1}\cdot {\left(\frac{1+(\gamma_{2}/\gamma_{1}\cdot C_{\nu 2}/C_{\nu 1}-1)\cdot y}{\left[1+(C_{\nu 2}/C_{\nu 1}-1)\cdot y]\cdot [1+(M_{2}/M_{1}-1)\cdot y\right]}\right)}^{0.5} \end{array}$$

where *f*_1_ is the oscillation frequency in neat gas of component 1, *M_i_*, γ*_i_*, and *C*_ν*i*_, *i* = 1, 2, are, respectively, molecular weights, adiabatic constants, and molar isochoric heats of the mixture components, and *y* is the concentration of component 2. The corresponding dependencies, calculated for CO_2_/N_2_ and H_2_/N_2_ gas mixtures using the data from the literature and the value of *f*_1_ derived from the experiment in neat N_2_, are plotted in Figure 5b by the dashed lines. These dependencies can be employed as the calibration curves to find the value of the unknown concentration.

### Buffer Gas Pressures, Temperatures, and Mass Densities

**Figure 6.**
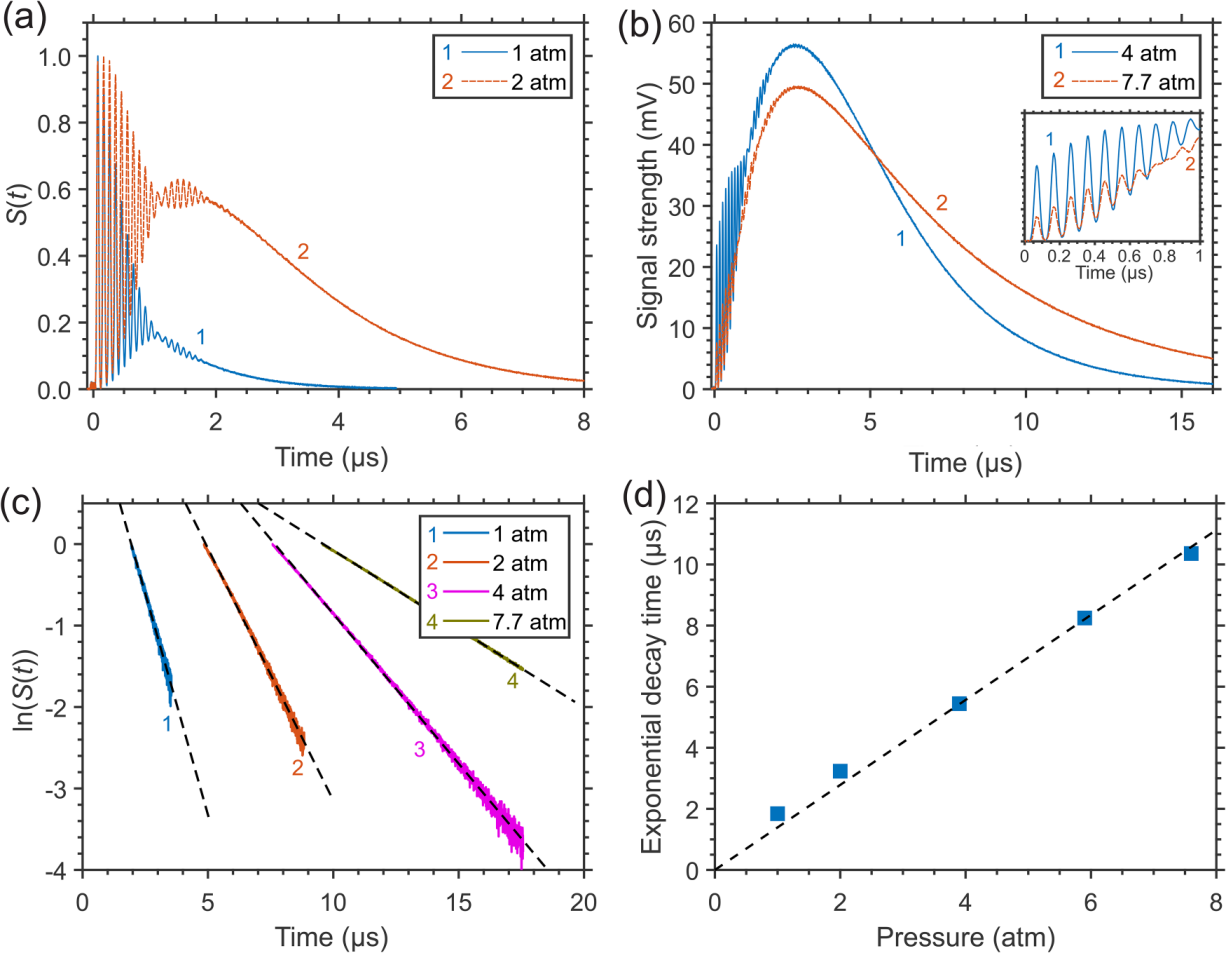
LITG signals generated in N_2_ at constant CH_4_ partial pressure (7.5 Torr) and *T* = 299 K: (a) at 1.0 and 2.0 atm, normalized by the first oscillation peak amplitude, or (b) at 4.0 and 7.7 atm, normalized by the amplitude of the hump, and the signal characteristics: (c) profile decays versus gas pressure (dashed lines: linear fits of the decays); (d) thermal diffusivity times, derived from the signal decay rates, versus gas pressure (dashed line: calculation).

The interference of the induced variations Δ*n* of the refractive index due to different collisional RET and VET processes in CH_4_ molecules is most pronounced in LITG signal profiles at higher pressures (or number densities) of a gas. As an example, Figures 6a and 6b present, for comparison, the LITG profiles produced, using the *R*(4) absorption line of CH_4_, in N_2_ with the same small CH_4_ partial pressure (7.5 Torr) at pressures 1.0, 2.0, 4.0, and 7.7 atm and *T* = 299 K. The profiles were recorded with the accumulation of 500 single-shot signals. The profiles at 1 and 2 atm are normalized by the first oscillation peak amplitude.

At 4.0 and 7.7 atm, the dominant contribution to the signal strength is due to the stationary gas density modulation. It results from the large energy release in the process of “slow” *V–T* relaxation of vibrationally excited CH_4_ molecules in the ν_4_ state in collisions with N_2_ molecules with the characteristic time τ*^V^*^–*T*^ ≈ 12.5 μs × atm (or ≈20 μs × atm).^40,51^ At constant partial pressure of CH_4_ both the signal strength and its integral over a definite delay time interval enhance with the increase of N_2_ pressure due to the growth of the stationary contribution. The growth of the signal strength is because a larger part of the internal energy obtained after absorption of laser radiation by CH_4_ molecules is released periodically in space before this periodic distribution of the excited molecules is destroyed by their mass diffusion. This diffusion (with the characteristic time τ*_D_*) becomes slower at higher mass density, or pressure (see Eq. 3). The stationary density modulation amplitude grows with pressure as a result of the variation of the characteristic LITG evolution time τ_s_ = (1/τ*_D_* + 1/τ*^V^*^–*T*^)^–1^ and the respective increase of the ratio τ_s_/τ*^V^*^–*T*^ = (1 + τ*^V^*^–*T*^/τ*_D_*)^-1^, which defines this amplitude.^25^ In addition, the created modulation of the refractive index decays slower, with a larger time τ_th_ proportional to mass density, or pressure (see Eq. 2). As a consequence, the sensitivity of the LITG response to the presence of a given amount of CH_4_ molecules in the buffer gas increases with its pressure. Meanwhile, as seen from Figures 6a and 6b, the acoustic contribution to the signal strength due to the rapid energy release in the process of “instantaneous” and “fast” *R*,*V–R′*,*V′* relaxation decreases compared to the stationary one at higher pressures.

Note that on the absolute scale in Figure 6b, the amplitudes of the first oscillation peaks at 4 atm are about three times greater than at 7.7 atm. This is clearly visible in the inset, where these oscillations are presented in more detail, at higher temporal resolution. At the same time, the amplitudes of the signal humps at these pressures are comparable. This does not contradict what was mentioned above, since a smaller amplitude of signal oscillations at higher pressure, with the same partial pressure of CH_4_, corresponds to a smaller number of excited molecules due to the smaller absorption coefficient in the center of the pressure-broadened line. At the same time, this smaller number of excited molecules provides, as explained above, a larger amplitude of the stationary contribution at higher pressures.

Noteworthy is the resumption of small regular signal oscillations in the interval of delays ≈0.8–1.8 μs, especially clearly noticeable in Figure 6a in the signal at 2 atm against the background of a large stationary contribution. This resumption of oscillations is also discernible both at atmospheric pressure of buffer gases (Figure 6a, Figure 1 and Figure 2) and at higher pressures (Figure 6b and Figure 3). The oscillations appear to be associated with the reappearance and then decay of a standing acoustic wave in the probe volume. A similar effect was clearly observed for LITG signals, e.g., in neat O_2_,^25^ as well as in mixtures of C_2_H_2_ with Ar, N_2_, and air.^33^ The possible nature of the effect was discussed by Sahlberg et al.,^33^ with the conclusion that it most likely results from the generation of two slightly different gratings due to a specific mode structure and spatial profile of the pump radiation, while the gratings are probed with a beam of smaller diameter than the diameter of the pump beam. However, since the resumption of oscillations was observed in a few independent experiments with different pump and probe lasers and different gases, its origin appears to be more physical. It can be assumed that the effect is the result of partial back reflection of traveling acoustic waves from density gradients near the pump beam boundary due to stronger heating of the gas by the unmodulated part of its radiation on the axis of the probe volume. In this case, the presence of a stationary density modulation with a large amplitude will emphasize the contribution of a secondary acoustic density modulation with a relatively small amplitude to the signal *S*(*t*) ~ (Δ*n*)^2^ due to their addition, which determines the resulting overall variation of the refractive index Δ*n*, and the appearance of a cross term when Δ*n* is squared. However, the small effect discussed does not affect the determination of oscillation frequencies and decay times carried out here and is not considered when analyzing the presented data.

The analysis of the LITG signal temporal profiles recorded at different buffer gas pressures (1–8 atm) under isothermal conditions has confirmed the high sensitivity of these profiles to variations of pressure and, thus, the feasibility of defining pressure from the signal shape. This can be done by deriving the decay time τ_th_ of the LITG signal due to heat conduction, which is determined, in accordance with Eq. 2, by pressure-dependent gas thermal diffusivity. The value of τ_th_ can be obtained either by fitting the whole signal profile *S*(*t*) using an appropriate collisional RET and VET model, following^25^ or, more directly, by measuring the gradient of the ln(*S*(*t*)) dependency, in a similar way as was done by Willman et al.,^13^ but in the signal tail, determined by the stationary contribution. However, it should be noted that a LITG signal profile with the stationary contribution due to “slow” collisional relaxation can be represented at large delays in a simple form *S*(*t*)  ∼ exp(–2*t*/τ_th_), which directly gives τ_th_ and, hence, buffer gas pressure, only if the relation τ_s_ << τ_th_ for the characteristic LITG evolution time τ_s_ is valid. This relation is fulfilled the better, the higher the pressure. The lower pressure limit increases approximately linearly with temperature due to changes in buffer gas mass density ρ ∝ 1/*T* and is also determined by the temperature-dependent molecular properties of the gas, which define the parameters τ*^V^*^–*T*^, τ*_D_*, and τ_th_.

The linear fitted ln(*S*(*t*)) tails of the LITG signals in Figures 6a and 6b are shown in Figure 6c. The inverse slopes of these dependencies, proportional to the signal exponential decay time, were used to calculate the values of this parameter plotted in Figure 6d. There are no error bars here because they are contained within the symbols. The derived values grow linearly with gas pressure and, in addition, lie on a straight line with the slope of 1.34 μs atm^−1^ corresponding to τ_th_ calculated at Λ = 34.1 μm from the thermal diffusivity of N_2_ at *T* = 299 K and 1 atm. Hence, the linear dependency obtained can be easily used for pressure measurements at a given temperature. Note that at the lowest pressure of 1 atm, there is a small deviation of the derived decay time from the calculated value of τ_th_ that is not present at higher pressures, for the reasons described above.

The recordings of the LITG signal profiles at different temperatures in the range of 26–474 °C (299–757 K) were performed under isobaric conditions (at ambient pressure) in slow laminar gas flows of N_2_ with 1% of CH_4_, directed through an open heating gas tube, at a given temperature. LITGs were generated at the peak of the multiplet *R*(6) line of the ν_3_ band of CH_4_ at 3086.0 cm^–1^. The signal oscillation frequency at a given Λ is defined by the sound velocity dependent on the local temperature. The temperatures were determined by fast Fourier transform of the signal profiles to derive oscillation frequencies *f*, as was commonly done previously by, e.g., Roshani et al.^17^ and Kozlov et al.^18^ Then, temperatures were calculated based on the corresponding frequencies, assuming that the fringe spacing Λ is known, the gas composition is known (N_2_), and the adiabatic sound velocity υ_s_ is related to temperature *T* by the known relation for an ideal gas (used to obtain Eq. 4):

(5) $$v_{s}=\Lambda \cdot f=\sqrt{\gamma \cdot \left(\frac{R_{m}}{M}\right)\cdot T}$$

where *M* and γ are the molecular weight and the adiabatic constant of the gas, and *R*_m_ is the universal gas constant. Temperature dependence of γ was neglected in the selected temperature range. A calibration measurement at a known temperature *T*_0_ was used to determine the values of *f*_0_ and Λ, as it was done by, e.g., Brown and Roberts,^5^ Stampanoni-Panariello et al.,^6^ Kozlov et al.,^7^ and Hemmerling et al.^21^ Alternatively, the temperatures can be calculated using the relation *T* = *T*_0_·(*f*/*f*_0_)^2^.

**Figure 7.**
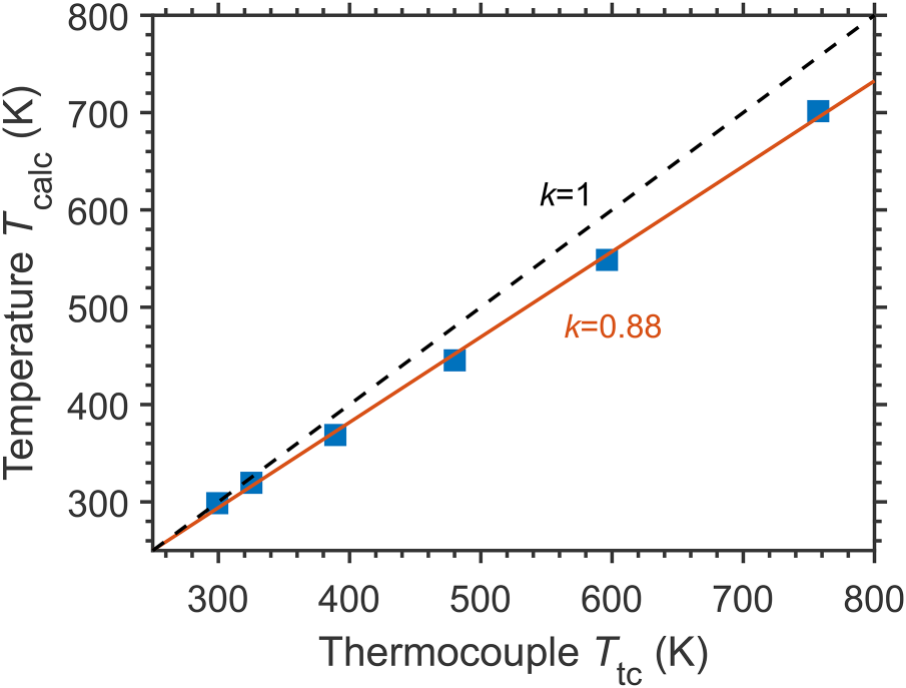
Local gas temperatures derived from the LITG signal profiles at ambient pressure: correspondence between gas temperatures *T*_calc_ and the readings of the thermocouple *T*_tc_ (solid squares: experimental data; red solid line: linear fit; black dashed line shows the relation *T*_calc_ = *T*_tc_.

The results of the measurements in a heating tube are presented in Figure 7, where the temperatures *T*_calc_, derived from the LITG signal profiles, are compared to the readings of the thermocouple *T*_tc_ located near the probe volume. The precision of temperature determination is about ±1 K for temperatures close to ambient temperature and ±10 K for the highest ones. These numbers are common for well-defined gas compositions during the measurements. The changes in *T*_calc_, as related to the reference temperature (*T*_0_ = 299 K), increase linearly with those in *T*_tc_: (*T*_calc_ – *T*_0_) = 0.88·(*T*_tc_ – *T*_0_). The thermocouple, placed a bit closer to the hot wall of the tube than the LITG probe volume, gives slightly higher values compared to the LITG temperatures. The results expectedly show a good sensitivity of the LITG signal profiles to temperature, which provides the possibility of local gas thermometry up to high temperatures of about 1500–1800 K, characteristic for CH_4_ pyrolysis, as well as biomass, associated gas, and municipal waste combustion.

The signal oscillation frequency and decay time in the tail can be independently derived from its profile. Since sound velocity in a gas is weakly dependent on pressure, the oscillation frequency can be used for the calculation of the local temperature at any pressure, if the gas composition is known. In addition, at a known pressure, the local mass density of the gas can be derived.

Gas thermal conductivity and heat capacity are temperature-dependent. With the *T*-dependency of these parameters taken from the literature, the thermal diffusivity can be approximated by a polynomial to evaluate, at the fast Fourier transform-derived temperature, the value of τ_th_ at a known pressure. In our case, these values for the signals in heated N_2_ at 1 atm were calculated at the temperatures presented in Figure 7 and compared to the exponential decay times derived from the gradients of the ln(*S*(*t*)) dependencies, as described above. Unlike the values in Figure 6d, these decay times noticeably overestimate the corresponding values of τ_th_ and, in addition, do not follow their temperature dependency. The reason for this is that with increasing temperature at a given pressure, the gas density decreases according to the ideal gas law, and the relation τ_s_ << τ_th_ becomes invalid. Therefore, care should be taken when using directly determined exponential decay times of the signals in N_2_ at higher temperatures to derive gas pressure, similar to how it was used at ambient temperature.

## Conclusion

The presented results demonstrate that resonant mid-IR (3.3 μm) spatially periodic excitation of CH_4_ molecules in the region of the strong ν_3_ fundamental absorption band, producing LIGs of the refractive index in CH_4_-containing buffer gases, such as Ar, N_2_, H_2_, and CO_2_, causes strong LITG signals even at low concentrations of CH_4_. The reason for this is the fast collisional release of a large amount of the internal energy of laser-excited CH_4_ molecules to the environment.

The large strength of LITG signals enables local diagnostic measurements, also single-shot, which simultaneously provide several selected parameters of a gas medium. As a result, low-concentration CH_4_ molecules originally present in many gaseous media can be employed as effective probes that do not affect the properties of the mixture.

The presence of numerous IR-active ro-vibrational transitions with a wide range of absorption line strengths in the ν_3_ band of CH_4_ makes easier the task of narrowband selective and efficient excitation of these molecules between absorption lines of other molecules in the gas. This enables selectivity of CH_4_ concentration measurements and of probing the mixture parameters.

The strength of the registered LITG signals, as well as their characteristic and developed temporal profiles, are strongly dependent on CH_4_ concentration, mixture composition, gas pressure, and temperature. This opens prospects for obtaining these parameters. Examples are given of how the registered LITG signals, resulting from resonant excitation of CH_4_ molecules in the mid-IR range, can be employed to determine CH_4_ concentration and CH_4_-containing gas mixture characteristics by using simplified methods of data evaluation and calibration curves. This makes it possible to avoid complicated simulations of the temporal profile of the signals, which considers the paths of molecular collisional relaxation and transition rates. Manifestations of the pump radiation absorption in the gas at higher CH_4_ concentrations are demonstrated and discussed.

To conclude, the presented results show good potential for LIG signal profiles in CH_4_-containing gas mixtures for measurement of different gas parameters, such as gas composition, pressure, and temperature.
